# Cone beam computed tomography and artificial intelligence. ¿Where we are?

**DOI:** 10.21142/2523-2754-1204-2024-214

**Published:** 2024-11-23

**Authors:** Heraldo Luís Dias da Silveira

**Affiliations:** 1 Department of Oral Surgery and Orthopedics, Division of Dental Radiology, Dental School, Federal University of Rio Grande do Sul (UFRGS). Porto Alegre, Rio Grande do Sul, Brazil. heraldo.silveira@ufrgs.br Universidade Federal do Rio Grande do Sul Department of Oral Surgery and Orthopedics, Division of Dental Radiology Dental School Federal University of Rio Grande do Sul (UFRGS) Porto Alegre, Rio Grande do Sul Brazil heraldo.silveira@ufrgs.br

In recent years we have seen a huge number of papers presenting the different possibilities for artificial intelligence (AI) in dentistry. Within the next decade, AI will transform the workflow of modern dental practice. Additionally, AI tools can enable healthcare professionals to increase their reliability, effectiveness, and usefulness, nonetheless potential limitations and errors. ^1^

Systematic reviews concerning artificial intelligence for radiographic imaging detection of caries lesions concluded that AI-based models exhibit good diagnostic performance. AI provides comprehensive, reliable, and accurate image assessment and disease detection, thus facilitating effective and efficient diagnosis and improving the prognosis of dental caries in periapical radiographs. However, the various AI models come with their own set of pros and cons, so the selection of a model should be based on the specific task objectives and requirements. ^(2, 3)^ Accordingly, research projects should be meticulously designed to detect the true performance of AI models.

In this context, we may hope that AI technology, merged with Cone beam computed tomography (CBCT), will be routinely used in 3D diagnostic in modern dental practice. Diagnosis made by radiologists on cone beam computed tomography (CBCT) in dentistry has been an area of great interest for applying artificial intelligence (AI). 

AI, particularly convolutional neural network (CNN) models, has been applied to identify and diagnose conditions in medical images, including cone beam CT scans. Research show that AI systems can detect dental fractures, bone lesions, cysts, abscesses, and other pathologies with high accuracy, often comparable to that of experienced radiologists. ^4^ Integrating artificial intelligence and CBCT imaging has the potential to speed up the dental workflow, streamline oral healthcare, and facilitate preventive dentistry. ^5^

AI has been seen as a complementary tool to conventional diagnostics, being able to quickly analyze large volumes of images and highlight areas of interest for radiologists, facilitating decision-making. ^6^ This is particularly useful in practices with large scan volumes or for specialists with less experience with complex images.

Interpreting medical images is not a purely technical task; it often relies on a deep understanding of the patient’s clinical context and history. While AI can detect patterns in images, it does not have the same ability to assess clinical context as a human radiologist. Despite good results in specific studies, AI may not generalize well to all clinical scenarios or types of patients. Validating AI systems on large, diverse datasets remains a challenge. ^7^

Although AI has shown great potential, it does not completely replace human diagnosis in cone beam CT scans. Still, it can significantly assist by improving efficiency, accuracy, and early detection of pathologies.
